# Potential drug-related problems detected by routine pharmaceutical interventions: safety and economic contributions made by hospital pharmacists in Japan

**DOI:** 10.1186/s40780-018-0125-z

**Published:** 2018-12-13

**Authors:** Yuichi Tasaka, Akihiro Tanaka, Daiki Yasunaga, Takashige Asakawa, Hiroaki Araki, Mamoru Tanaka

**Affiliations:** 1 Division of Pharmacy, Ehime University Hospital, 454 Shitsukawa, Toon, Ehime 791-0295 Japan; 2 Ehime Society of Hospital Pharmacists, 454 Shitsukawa, Toon, Ehime 791-0295 Japan; 3 Department of Pharmacy, Saiseikai Saijo Hospital, 269-1 Tsuitachi, Saijo, Ehime 793-0027 Japan; 4 School of Pharmacy, Shujitsu University, 1-6-1 Nishigawara, Naka-ku, Okayama, Okayama 703-8516 Japan; 5 Division of Pharmacy, Ehime University Hospital, 454 Shitsukawa, Toon, Ehime 791-0295 Japan

**Keywords:** Pharmaceutical intervention, Adverse drug reaction, Potential drug-related problem, Elderly patients, Renal dysfunction, Polypharmacy

## Abstract

**Background:**

Pharmaceutical intervention enables safe and effective pharmacotherapy by avoiding of adverse drug reactions (ADRs) and efficacy attenuations. Many prescriptions require optimization, and pharmaceutical interventions are inextricably associated with the prevention of potential drug-related problems (DRPs). Although the analysis and understanding of pharmaceutical interventions can lead to improvement in prescription, the analysis of routine pharmaceutical interventions in Japan in insufficient. Thus, we conducted this study to understand potential DRPs by analyzing routine pharmaceutical interventions made by pharmacists in Japan.

**Methods:**

Pharmacists register the details of pharmaceutical interventions (excluding personal patient information) in a web-based database. We classified data of pharmaceutical interventions into 13 DRP types, 43 DRP subtypes, and 10 intervention categories (e.g., avoidance of serious ADRs and renal dosing recommendations). These data were analyzed with a focus on renal dysfunction and polypharmacy.

**Results:**

During the study period, 2376 pharmaceutical interventions were performed. Overall, 68.2% of pharmaceutical interventions were for patients aged over 65 years. The most frequently detected potential DRP was overdosage, followed by omission of prescription, contraindications, and duplication of a drug with similar effect. The main cause of contraindication and overdosage was renal function deterioration, and that of polypharmacy was duplication of a drug with similar effect. Using our original evidence-based approach, we found that 2376 pharmaceutical interventions prevented ADRs for 1678 drugs, with potential cost savings of up to USD 2,657,820.

**Conclusions:**

Our results indicate that the analysis of routine pharmaceutical interventions is beneficial for detecting potential DRPs. Our findings also show that, in an aging society, pharmacists have an important role in providing medication safety, with potential cost savings.

## Background

Pharmaceutical intervention enables prescription optimization and can prevent adverse drug reactions (ADRs) and efficacy attenuations, which are extremely important to providing safe and effective pharmacotherapy. A meta-analysis in the United States reported that the overall incidence of serious and fatal ADRs among hospitalized patients was 6.7 and 0.32%, respectively [1]. Multi-institutional joint research in Japan showed that adverse drug events (ADEs) occurred in 29 of 100 hospital admissions, of which 4.9 and 1.6% were serious and life-threatening, respectively [2]. Patients who experience ADRs have a higher mortality and a longer hospital stay than those who do not [3]. Furthermore, a retrospective study in Canada showed that a severe ADR has an associated cost of three times that of a mild ADR [4]. In fact, annual relief benefits paid by “the Relief System for Sufferers from Adverse Drug Reactions” in Japan 2017 amounted to over USD 20 million (1 USD = 100 JPY) [5]. Thus, avoiding preventable ADRs is important from the perspectives of both safety and cost, particularly with the increasing medical expenses associated with a rapidly aging society.

Studies have shown that half of ADRs are preventable [6–9]. An analysis of ADEs resulting in emergency hospitalizations in older patients at 58 institutions in the United States showed that approximately 65% of ADEs were caused by unintended overdosage, and that more than half of the affected patients had been prescribed at least five drugs [10]. Particularly in elderly patients, renal dysfunction is responsible for ADRs owing to unintended overdosage caused by the delayed excretion of many drugs (e.g., water-soluble antimicrobials, diuretics, and non-steroidal anti-inflammatory drugs [NSAIDs]) [11]. At the same time, polypharmacy additively increases the risk of ADRs, and drug–drug interactions increase this risk even further [12]. In fact, renal dysfunction and polypharmacy raise the hazard ratio of preventable ADRs by 2.6 and 2.7 times, respectively [7]. For these reasons, pharmaceutical interventions that reduce medication risk by dosage adjustment based on metabolic function and terminating stoppable drugs are vital to avoid preventable ADRs.

Using an original evidence-based approach, we previously showed that pharmaceutical interventions at university hospitals in Japan may save up to USD 228,160 per year by preventing ADRs [13]. Furthermore, we developed a web-based database of pharmaceutical interventions, and illustrated the characteristics and problems of intervention by community and hospital pharmacists [14]. However, the underlying problems of the prescriptions requiring pharmaceutical interventions were not fully elucidated in our earlier study. Many potential prescriptions require optimization, and pharmaceutical interventions appear to be inextricably associated with the avoidance of drug-related problems (DRPs). In other words, the analysis of routine pharmaceutical interventions can be used to detect potential DRPs, leading to improvement in prescription. Thus, in this study, we analyzed routine pharmaceutical interventions to reveal potential DRPs in Japan’s aging society. Furthermore, as our previous studies were conducted in only one or two hospitals [13–15], here we examined potential DRPs based on pharmaceutical interventions at 20 hospitals in Ehime Prefecture, Japan.

## Methods

### Definition of pharmaceutical intervention and polypharmacy

In this study, we defined a pharmaceutical intervention by a hospital pharmacist as a change in a prescription or test order for inpatients or outpatients as the result of a query about the prescription or a consultation at the hospital. Polypharmacy was defined in this study as the receipt of more medication than is necessary. Thus, pharmaceutical intervention to prevent the administration of unnecessary medication was defined as pharmaceutical intervention against polypharmacy.

### Collection of data associated with pharmaceutical interventions

This study was performed at 20 hospitals from April 2015 to March 2017 in Ehime Prefecture, Japan. Data on pharmaceutical interventions were stored in a web-based database that we previously developed using FileMaker Server 13 v3 [14]. After performing a pharmaceutical intervention, pharmacists spontaneously and anonymously uploaded the details of the intervention to the database, excluding the patient’s personal information (e.g., name, date of birth, and address). The pharmacists did not receive any special training for this study, except instructions on how to use the database. Pharmaceutical interventions registered by pharmacists were assigned to specific DRP types/subtypes and pharmaceutical intervention categories as described below.

### Categorization of potential DRP types and subtypes

In this study, potential DRPs were defined as pharmaceutical issues in prescriptions that were detected by pharmacists and pharmaceutical issues and concerns identified to pharmacists by physicians. Specifically, potential DRPs were considered as DRPs of which physicians were not aware, or that were not resolved until pharmacist intervention. We defined our original potential 13 DRP types and 43 subtypes by referring to Hepler-Strand classification, with modifications [16, 17]. The 13 DRP types were as follows; Improper drug selection; Drug interaction; Overdosage; Subtherapeutic dosage; Inappropriate route selection; Improper dosing timing; Drug use requiring therapeutic drug monitoring (TDM); Poor compliance; Untreated/undertreated indications; Lack of monitoring by examination; Adverse drug reactions; Consultation from doctor; and Other. After analyzing the details of pharmaceutical interventions, each intervention was assigned to a potential DRP subtype (Table 2) on a one-to-one basis.

### Estimation of economic impact of pharmaceutical intervention

Pharmaceutical interventions were classified in accordance with previous studies [13–15]: Avoidance of serious ADRs; Transvenous antimicrobial therapy interventions; Interventions concerning cancer chemotherapy; Avoidance of drug interactions; Renal dosing recommendations; Avoidance of intravenous drug incompatibility; Confirmation of medication history; Drug therapy consultations and recommendations (non-renal/extensive); Monitoring recommendation; and Prescription term adjustment until the next consultation day. Estimation of economic impact was performed using our original evidence-based approach [13–15], that is, the economic impact of pharmaceutical interventions was estimated as the association with avoidance of ADRs. In Japan, the Pharmaceutical and Medical Devices Agency (PMDA) provides information about costs associated with harm to health when drugs have been used correctly (e.g., diseases and disabilities requiring hospitalization caused by adverse reactions to drugs prescribed at hospitals and clinics). The cost savings per case resulting from the avoidance of serious ADRs is approximately USD 21,400 (using an exchange rate of 1 USD = 100 JPY), based on the mean amount paid by the compensation (benefit/insurance) system. We also determined the economic impact of other routine pharmaceutical interventions, using the rate at which a routine intervention actually prevents an ADR [18]. We divided the rate (2.6–5.21%) into three grades according to the frequency of ADRs. Interventions involving cancer chemotherapy most frequently involve ADRs and had an impact of USD 1120 (USD 21,400 × 5.21%); avoidance of drug interactions, renal dosing recommendations, avoidance of intravenous drug incompatibility, confirmation of medication history, drug therapy consultations and recommendations (non-renal/extensive) had an impact of USD 840 (for high-risk drugs, involving more frequent ADRs [21,400 × 3.91%]) and USD 560 (for other drugs with a normal risk of ADRs [21,400 × 2.6%]). Drugs defined as high risk included immunosuppressants, antiarrhythmic drugs, anticonvulsants, anticoagulants, digitalis and digitalis preparations, anti-HIV drugs, theophylline preparations, injectable potassium preparations, and psychotropic and antidiabetic agents. A previous study reported that the economic impact of antimicrobial stewardship, that is, promotion of the appropriate use of antimicrobial injections, was 27,237 JPY / patient / day at a university hospital in Japan [19]. Thus, we assigned a value of USD 1900 (USD 272.37 × 7 [mean duration of administration of anti-MRSA drugs in Ehime University Hospital]) to transvenous antimicrobial therapy interventions that result in the avoidance of ADRs and efficacy attenuation, and improvements in pharmacotherapy.

Monitoring recommendations were not assigned a value because prescriptions were not changed by this intervention. Adjustment of the prescription term to the next consultation day was also assigned a value of USD 0 because this intervention may have been caused by the patient or the patient’s family and not the pharmacist. In this study, we did not assign any economic impact to interventions that might contribute to an improvement in pharmacotherapy (e.g., avoidance of efficacy attenuation) and patients’ quality of life (QOL) (e.g., suggestions for pain control), except for transvenous antimicrobial therapy.

## Results

### Characteristics of patients receiving pharmaceutical interventions

During the study period, 2376 patients received pharmaceutical intervention by hospital pharmacists. In this study, 84.5% of patients were inpatients, and 14.4% were outpatients (Table 1a). The age group of patients who most frequently received pharmaceutical intervention was 80–89 years (24.8%), followed by 70–79 years (24.7%) and 60–69 years (20.5%). Of note, among all patients, 68.2% were older than 65 years old, and 42.3% were older than 75 years old (Table 1b). The median age of patients receiving pharmaceutical interventions was 72 years (interquartile range [IQR]: 61.0–81.0). The sex ratio of patients was approximately equal (males 51.1%, females 48.6%, and unknown 0.3%) (Table 1c).

**Table 1:** Characteristics of patients intervened by pharmacists, according to a) category, b) age, and c) sex

	Number and percentage of cases	
a) Category		
Inpatient	2008	84.5%
Outpatient	341	14.4%
Unknown	27	1.1%
Overall	2376	100%
b) Age (years)		
0–9	90	3.8%
10–19	37	1.6%
20–29	31	1.3%
30–39	61	2.6%
40–49	134	5.6%
50–59	187	7.9%
60–69	487	20.5%
70–79	588	24.7%
80–89	589	24.8%
90+	153	6.4%
Unknown	19	0.8%
Overall	2376	100%
65+	1621	68.2%
75+	1005	42.3%
c) Sex		
Male	1213	51.1%
Female	1155	48.6%
Unknown	8	0.3%
Overall	2376	100%

### Potential DRPs detected by routine pharmaceutical interventions

**Fig. 1:**
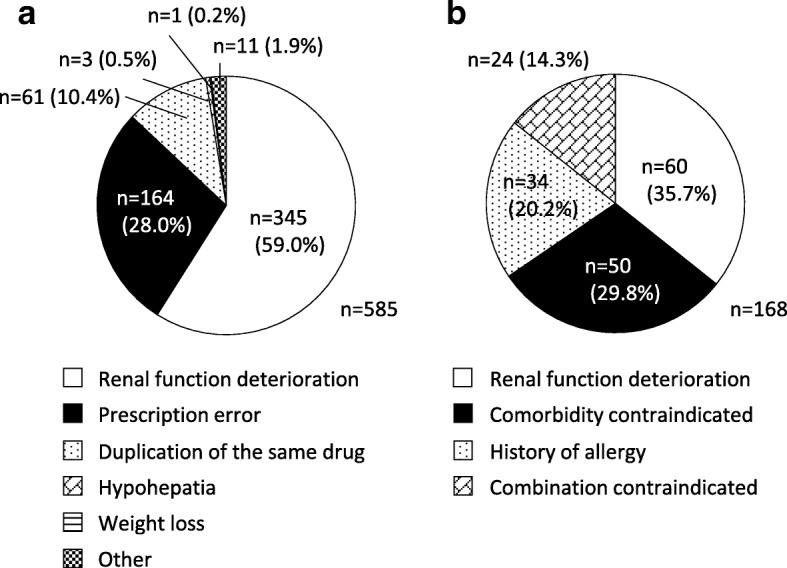
Causes of avoided contraindications and overdosage. **a**) overdosage, **b**) contraindications

Table 2 shows the types, subtypes and number of potential DRPs detected by routine pharmaceutical intervention. The most common type of DRP was Overdosage (*n* = 608, 25.6%). Overall, 585 overdosages were detected, representing the most frequent DRP subtype, followed by omission of prescription (*n* = 166), contraindication (*n* = 144), duplication of a drug with similar effect (*n* = 140), and underdosage (*n* = 129). The causes of overdosage included renal function deterioration (*n* = 345, 59.0%), prescription error (*n* = 164, 28.0%), duplication of the same drug (*n* = 61, 10.4%), hypohepatia (n = 3, 0.5%), weight loss (n = 1, 0.2%), and other (*n* = 11, 1.9%) (Fig. 1a). The median age of patients requiring pharmaceutical interventions against overdosage caused by renal dysfunction was 80 years old (IQR: 72.0–87.0). Furthermore, 144 contraindications and 24 contraindications for co-administration were detected (Table 2); the main cause of contraindication was also renal dysfunction (median age of patients was 82 years [IQR: 75.5–90.0]) (Fig. 1b). Drugs that could be increased or decreased according to the package insert were included in “overdosage” and “underdosage” DRPs. Pharmacist interventions were based on guidelines or medication effects and resulted in the avoidance of ADRs or improvements in pharmacotherapy.

**Table 2:** Potential drug-related problems detected by routine pharmaceutical interventions

Potential drug-related problems, type and subtype	Number of cases
Improper drug selection	
Prescribing the wrong drug	30
Duplication of drug with similar effect	140 (4)
Contraindications (except drug interaction)	144 (3)
Careful administration	42
Use during pregnancy, delivery, or lactation (except contraindications)	2
Prescription of discontinued drug	30
Administration of stoppable drug	70
Drug interaction	
Contraindications for co-administration	24
Precautions for co-administration	61
Overdosage	
Overdosage	585 (1)
Dosing rate too fast	15
Dilute concentration too high	8
Subtherapeutic dosage	
Underdosage	129 (5)
Dosing rate too slow	11
Dilute concentration too low	5
Inappropriate route selection	
Intravenous drug incompatibility	
Inappropriate solvent	26
Inappropriate route	26
Inappropriate use of filter	2
Improper dosing timing	
Inappropriate dosing time	55
Lack of rest period	12
Drug use requiring therapeutic drug monitoring	
Initial dose setting	43
Dose setting (except initial case)	63
Poor compliance	
Overuse	12
Underuse	1
Does not take medicine	15
Taking a cancelled drug	4
Inappropriate crushing or dissolution of tablets	68
Difficulty using dosage form	60
Untreated/undertreated indications	
Necessary medication not started/restarted	117
Omission of prescription	166 (2)
Condition undertreated	39
Drug must be discontinued before a test/surgery	24
Stopping a drug that should be continued	18
Lack of monitoring by examination	
Necessary examination not performed	82
Adverse drug reactions	
Presence of serious adverse drug reactions	40
Presence of other adverse drug reactions	99
History of serious adverse reactions	8
History of other adverse drug reactions	28
Consultation from doctor	
Drug selection	10
Dosage selection	16
Drug and dosage selection	20
Other	6
Other	
Other	20
Overall	2,376

( ) is order of the top five potential DRP subtypes

### Renal dosing recommendations and test results used by pharmacists

During the study period, 436 renal dosing recommendations and dosage/usage optimizations were performed, including 39 transvenous antimicrobial therapy interventions, and 12 interventions concerning cancer chemotherapy. As a result of these interventions, the dosage/usage of 84 types of drugs was adjusted in accordance with patients’ renal function. Table 3a shows the top 10 drugs that required dosage/usage adjustment; the most common drug was levofloxacin, followed by cefcapene pivoxil and famotidine. In addition, 21 drugs had a contraindication for reasons of renal dysfunction, and most of these were high-risk drugs such as edoxaban, metformin, and apixaban (Table 3b). Furthermore, 627 of 2270 pharmaceutical interventions (except for TDM requiring renal function and drug blood level) were performed based on test results; 448 (71.5% of 627) pharmaceutical interventions were performed based on renal function (e.g., creatinine, creatinine clearance, and estimated glomerular filtration rate [eGFR]). Other common test results included markers associated with hepatitis B infection (e.g., hepatitis B surface antigen, hepatitis B core antibody, hepatitis B virus DNA; *n* = 63, 10.0%), body weight (*n* = 13, 2.1%), serum potassium level (*n* = 11, 1.8%), blood neutrophil count (*n* = 10, 1.6%), prothrombin time-international normalized ratio (PT-INR) (*n* = 9, 1.4%), and serum magnesium level (n = 9, 1.4%).

**Table 3:** Details of renal dosing recommendations; a) top 10 of 84 intervened drugs, and b) top 5 of 21 drugs that were contraindicated

	Objective drug	Number of cases
a) top 10 of 84 intervened drugs		
1	Levofloxacin	70
2	Cefcapene pivoxil	52
2	Famotidine	52
4	Edoxaban	38
5	Allopurinol	9
5	Sultamicillin	9
7	Magnesium oxide	8
7	Cefmetazole	8
7	Metformin	8
7	Meropenem	8
7	Rivaroxaban	8
7	Levocetirizine	8
b) top 5 of 21 drugs that were contraindicated		
1	Edoxaban	22
2	Metformin	7
3	Apixaban	4
3	Eplerenone	4
3	Duloxetine	4

### Pharmaceutical interventions against polypharmacy made by pharmacists

**Fig. 2:**
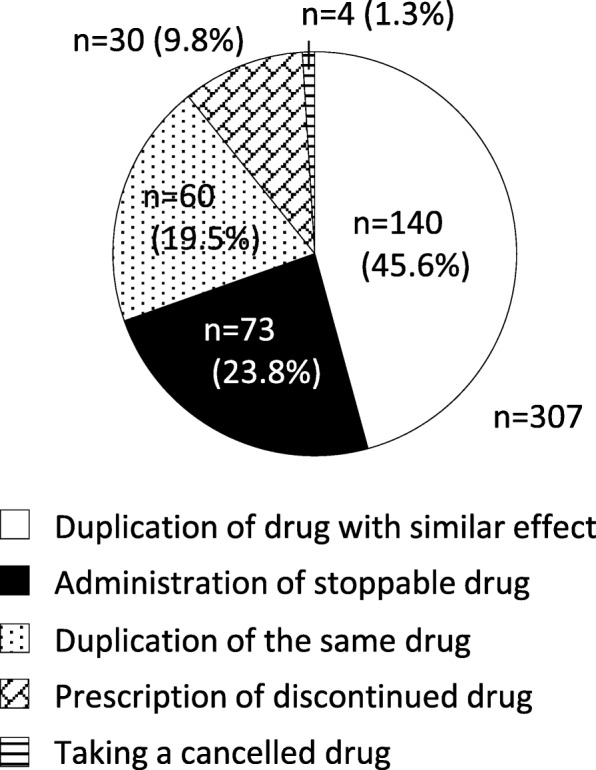
Main reasons for polypharmacy

A total of 307 interventions by pharmacists led to the resolution of polypharmacy during the study period. The most frequent reason for polypharmacy was duplication of a drug with a similar effect (*n* = 140, 45.6%). Other reasons were administration of a stoppable drug (including one case of presence of serious ADRs and two cases of difficulty using dosage form) (*n* = 73, 23.8%), duplication of the same drug (counted as overdosage) (*n* = 60, 19.5%), prescription of a discontinued drug (*n* = 30, 9.8%), and taking a cancelled drug (*n* = 4, 1.3%) (Fig. 2). Through these pharmaceutical interventions, a total of 38 classes of drug and a total of 320 drugs were discontinued; Table 4 shows the top five drug classes. Antiulcer drugs were the most frequently discontinued drugs (e.g., potassium-competitive acid blockers [P-CABs], proton pump inhibitors [PPIs], and H_2_ receptor antagonists), and were mainly detected as duplicate prescriptions of drugs with a similar effect. Pharmacists also intervened for antihypertensive drug preparations based on patients’ symptoms, resulting in discontinued prescription.

**Table 4:** Top five drug classes in which polypharmacy was avoided by pharmacist intervention

	Drug class	Number of cases
1	Antiulcer drug^a^	110
2	Antihypertensive	19
3	Anticoagulant / Antiplatelet drug	18
4	Antibiotics	17
5	Antidiabetic agent	16

^a^Potassium-competitive acid blocker, proton pump inhibitor, H_2_ receptor inhibitor, and other mucosal protectants

### Types of drugs requiring pharmacist intervention to avoid ADRs

During the study period, a total of 1678 drugs required pharmacist intervention to avoid ADRs. As a result, the most common drug type was anti-infectives, including antibiotics, antimycotics, and antivirotics (*n* = 388, 23.2%). Other common drug types were: anticancer agents (*n* = 292, 17.4%), antiulcer drugs (P-CABs, PPIs, H_2_ receptor antagonists, and other mucosal protectants) (*n* = 180, 10.7%), anticoagulant and antiplatelet drugs (*n* = 140, 8.4%), cardiovascular agents (antihypertensives, antiarrhythmics, and drugs for coronary heart disease) (*n* = 85, 5.1%), psychoneurotic agents (antidepressants, sleeping pills, antiepileptics, antipsychotics, dementia medication, and central analgesics [except opioids]) (n = 85, 5.1%), antidiabetic agents (*n* = 60, 3.6%), NSAIDs (*n* = 49, 2.9%), steroidal anti-inflammatory drugs (*n* = 35, 2.1%), laxative agents (*n* = 26, 1.6%), and allergy medications (n = 26, 1.6%) (Table 5).

**Table 5:** Top 10 drug classes in which adverse drug reactions were avoided by pharmacist intervention

	Drug class	Number of cases
1	Anti-infective^a^	388
2	Anticancer agent	292
3	Antiulcer drug^b^	180
4	Anticoagulant / Antiplatelet drug	140
5	Cardiovascular agent^c^	85
5	Psychoneurotic agent^d^	85
7	Antidiabetic agent	60
8	Non-steroidal anti-inflammatory drug	49
9	Steroidal anti-inflammatory drug	35
10	Laxative agent	26
10	Allergy medication	26

^a^Antimicrobials, antimycotics, and antivirotics

^b^Potassium-competitive acid blocker, proton pump inhibitor, H_2_ receptor antagonist, and other mucosal protectants

^c^Antihypertensives, antiarrhythmics, and drugs for coronary heart disease

^d^Antidepressants, sleeping pills, antiepileptics, antipsychotics, dementia medication, and central analgesics (except opioids)

### Pharmaceutical interventions for potential DRPs and their economic impact

Table 6 shows a classification of pharmaceutical interventions and the associated economic impact. A total of 1759 pharmaceutical interventions contributed to the avoidance of ADRs (transvenous antimicrobial therapy includes avoiding efficacy attenuation and improvement of prescription). The total cost savings associated with the 1759 pharmaceutical interventions for DRPs (Table 2) by hospital pharmacists was USD 2,657,820. The average economic impact was USD 1511.0 per case (USD 2,657,820 for the 1759 interventions). There were 56 instances of direct avoidance of serious ADRs using several approaches: e.g., observation of the patients’ symptoms after medication, confirmation of blood test results after medication, and confirmation of a history of serious ADRs (DRP subtype: precautions for co-administration, careful administration, necessary medication not started/restarted, necessary examination not performed, presence of serious ADR, presence of other ADR, and history of serious ADR). A total of 265 transvenous antimicrobial therapy interventions mostly included TDM of vancomycin (*n* = 96, 36.2%). In addition, recommendations for antimicrobials based on the results of bacterial culture and allergic history were included as transvenous antimicrobial therapy interventions. A total of 268 interventions concerning cancer chemotherapy, including 58 monitoring recommendations, avoided ADRs by preventing the omission of prescriptions (e.g., preventing omission of necessary antiemetics, *n* = 94, 35.1%), monitoring recommendations regarding reactivation of hepatitis B virus (*n* = 58, 21.6%), and recommendation for supportive therapy (e.g., enhancement of antiemetic therapy, n = 58, 21.6%). A total of 697 drug therapy consultations or recommendations, including 22 monitoring recommendations, resulted in the avoidance of ADRs by discontinuing unnecessary drugs (*n* = 256, 36.7%) followed by preventing prescription error (*n* = 224, 32.1%) and countermeasures against ADRs, except cancer chemotherapy (*n* = 117, 16.8%). Of note, 16.0% of pharmaceutical interventions overall (*n* = 379 of 2376) took place when pharmacists conducted a preliminary check of drugs that patients brought with them to the hospital.

**Table 6:** Classification and economic impact of pharmaceutical interventions

Intervention type	Number of cases of avoided ADRs	Cost savings per case (USD)	Cost savings (USD)
Avoidance of serious adverse drug reactions (ADRs)	56	21,400	1,198,400
Transvenous antimicrobial therapy interventions^a^	265	1900	503,500
Interventions concerning cancer chemotherapy	210	1120	235,200
Avoidance of drug interactions			
High-risk drugs	8	840	6720
Normal drugs	20	560	11,200
Renal dosing recommendations			
High-risk drugs	89	840	74,760
Normal drugs	296	560	165,760
Avoiding intravenous drug incompatibility			
High-risk drugs	0	840	0
Normal drugs	0	560	0
Confirmation of medication history			
High-risk drugs	21	840	17,640
Normal drugs	39	560	21,840
Drug therapy consultation/recommendation (non-renal/extensive)			
High-risk drugs	160	840	134,400
Normal drugs	515	560	288,400
Monitoring recommendation	80	0	0
Prescription term adjustment until next consultation day	0	0	0
Overall	1759		2,657,820

^a^Includes avoiding efficacy attenuation and improvement of prescription

## Discussion

To understand potential DRPs, knowledge of the pharmaceutical interventions performed in clinical practice is of significant importance. In this study, we analyzed 2376 pharmaceutical interventions conducted by hospital pharmacists at 20 hospitals in Japan. Most patients who received pharmaceutical intervention were inpatients; outpatients were limited to those receiving cancer chemotherapy. This is because hospital pharmacists in Japan are mainly involved with pharmacotherapy for inpatients, against a backdrop of the recent separation of dispensing and prescribing functions in Japan. The age distribution of patients who received pharmaceutical interventions was mainly those aged 80–89 years, and 68.2% of all patients were older than 65 years. Because most inpatients receiving medical care in Japan are aged 80–84 years, followed by those aged 85–89 years and 75–79 years [20], the patient age distribution of this study reflected well that of inpatients receiving medical care in Japan. With respect to the type of facility, most hospitals that participated in this study provide medical care for acute-stage illness. A total of 68.2% of patients who received pharmaceutical interventions were elderly (median age 72 years), representing an appropriate background against which to discuss potential DRPs in an aging society.

Overdosage was detected as a major potential DRP and accounted for 24.6% of potential DRPs overall in the analysis using our original categorization. For this reason, we analyzed the causes of overdosage and found that 59.0% of cases corresponded to a deterioration in renal function. Furthermore, the main cause of contraindication was also renal dysfunction. In Japan, 13.3 million people have chronic kidney disease (CKD), and the prevalence of CKD increases with older age [21]. In fact, the median patient age for overdosage and contraindication caused by renal function deterioration was 80 years and 82 years, respectively. Thus, the results obtained in this study represent the current status, in that many prescriptions for renally eliminated drugs and potentially nephrotoxic drugs remain unadjusted according to each patient’s renal function; if carried out, such medication adjustment would contribute to the avoidance of preventable ADRs through prescription optimization by hospital pharmacists. Antibiotics (e.g., levofloxacin and cefcapene pivoxil) represented the majority of drugs requiring dose adjustment by pharmacists, according to individual renal function. Moreover, in many prescriptions for high-risk drugs (e.g., anticoagulant and antiplatelet drugs), contraindication for renal dysfunction was avoided. Furthermore, in one case, the pharmacist intervened for a patient with renal dysfunction who developed hypermagnesemia caused by magnesium oxide; the intervention prevented the hypermagnesemia from becoming life-threatening in the patient. These results highlight the importance of verifying the patient’s renal function and optimizing prescription based on renal function when using renally eliminated drugs in elderly patients. In some cases, the pharmacist recommended the use of a PPI to decrease H_2_ receptor antagonist usage because of renal dysfunction. Studies show that the use of H_2_ receptor antagonists can cause delirium and cognitive function decline in elderly patients [22, 23]; therefore, switching to a PPI may be useful from this perspective. However, long-term use of PPIs is reported to increase the risk of *Clostridium difficile* infection, community-acquired pneumonia, and hip fracture [24–26]. Thus, the use of PPIs requires careful monitoring to minimize such issues. In this study, pharmacists often used test results of renal function, but results for hepatic function were used with lower frequency. This suggests that the use of test results for hepatic function may provide pharmacists with a greater opportunity to intervene in prescriptions.

The number of therapeutic drugs tends to increase in proportion with increased comorbidities, especially in elderly patients. A previous study showed that the odds ratio of ADR was significantly higher in elderly patients taking six or more drugs [27]. Thus, discontinuing stoppable drugs can lead to avoidance of preventable ADRs. In this study, approximately half of stoppable drugs intervened by pharmacists were duplicates of a drug with a similar effect. Many cases reported in this study appeared to be duplication of drugs that the patient brought with them to the hospital and drugs used in accordance with the clinical pathway after hospital admission (data not shown). For this reason, antiulcer drugs (e.g., PPIs and H_2_ receptor antagonists) represented those in which intervention could most often resolve duplication of drugs with similar effect or duplication of the same drug. In this study, some interventions led to the discontinuation of antihypertensive, anticoagulant, and antiplatelet drugs; long-term follow-up (whether the complaint is exacerbated or not) after intervention is especially important in the case of these drugs. However, most patients receiving pharmaceutical intervention in this study were inpatients; therefore, we were unable to sufficiently expand on this point. Future measures may be needed to improve cooperation between primary care physicians and community pharmacists. The Screening Tool of Older Person’s Prescriptions (STOPP) and Screening Tool to Alert doctors to the Right Treatment (START) criteria are currently used to detect potential errors of commission and omission in prescribing [28, 29]. Hamilton et al. reported that potentially inappropriate medications (PIMs) defined in the STOPP criteria were significantly associated with avoidable ADEs in older people [30], and Kimura et al. demonstrated the usefulness of STOPP criteria ver. 2 to detect PIMs in elderly Japanese inpatients at a university hospital [31]. At the same time, a study among outpatients conducted in the Netherlands reported that most DRPs in community-dwelling older patients were not associated with STOPP/START criteria [32]. As stated in the Methods, pharmacists did not receive any special training for this study. Thus, educating pharmacists about these criteria would create more opportunities for them to intervene and avoid potential DRPs and preventable ADRs.

The drug category that most frequently required intervention to avoid ADRs was anti-infectives (antimicrobials, antimycotics, and antivirotics), likely because many renal dose recommendations (including TDM) and drug selection recommendations based on allergy history were against anti-infectives. Anticancer agents were the next most frequently intervened category, because the reason being that hospital pharmacists are actively involved in cancer chemotherapy, which is associated with a high frequency of ADRs. A prospective observational study reported that the most common class of drugs responsible for causing ADRs was anti-infectives, followed by steroids, anticoagulants, NSAIDs, and diuretics [33]. Our results support this finding, and it is therefore reasonable that pharmacists mostly intervened for anti-infectives in the present study. However, there may be additional opportunities to avoid ADRs caused by diuretics, which were less intervened in this study. In the present work, 16.0% of pharmaceutical interventions were performed upon a preliminary check of the drugs that patients brought with them to the hospital (nearly always conducted at the time of admission). This means that 16.0% of pharmaceutical interventions can potentially be performed by community pharmacists on an outpatient basis. Although the different backgrounds of how such pharmaceutical interventions can be performed (e.g., how to obtain test results and precise purpose of prescriptions) should be considered, sharing these results with community pharmacists may be important in the future.

The economic impact of pharmaceutical interventions by hospital pharmacists for several potential DRPs amounted to a total of USD 2,657,820, with an average cost savings of USD 1511.0 per case. Verdoorn et al. reported that 3.6 potential DRPs per patient were detected among prescriptions in elderly patients over 65 years taking more than five drugs [32]. Kimura et al. reported that 58.8% (483/822 patients) of elderly inpatients over 65 years were prescribed over seven drugs [31]. In addition, 74.0% (1759/2376) of pharmaceutical interventions for potential DRPs contributed to the avoidance of ADRs in this study. Considering the number of inpatients over age 65 years in Japan (937,300 patients) [20] and the proportion prescribed more than seven drugs, the economic impact associated with pharmaceutical interventions by hospital pharmacists that contribute to avoiding ADRs could reach over USD 2200 million (937,300 × 0.588 × 0.740 × 3.6 × 1511.0 = 2,218,475,454) in Japan. Because this value only represents elderly patients over age 65 years taking more than seven drugs, the economic contribution of hospital pharmacists associated with avoiding ADRs can be expected to be higher in practice. Niwa et al. described a review system for checking prescription in all patients receiving antimicrobial injections according to the intervention and feedback may save up to JPY 300 million (USD 3 million) per year at a university hospital in Japan [19]. Kato et al. showed that the cost-saving effect of improvements in pharmacotherapy, namely pharmaceutical interventions according to guidelines to decrease the risk of hospital readmissions, was JPY 1.5 million (USD 15,000) per year at only the cardiovascular ward of one university hospital in Japan [34]. Furthermore, pharmaceutical interventions leading to improve patient QOL are not estimated economic impact in the current study as far as we know. Therefore, the total economic contribution of intervention by hospital pharmacists on DRPs is likely to greatly exceed our estimates.

In summary, we detected potential DRPs by analyzing routine pharmaceutical interventions in this study. Pharmaceutical interventions at 20 hospitals were analyzed, and each hospital tended to have similar DRPs (data not shown). This suggests that the potential DRPs detected in this study may occur anywhere where there is a large aging population. Better understanding of potential DRPs, which currently causing problems that should be intervened, can lead to establishment of systems to prevent such DRPs from occurring. In other words, analysis of actual routine pharmaceutical interventions can to lead to a “Kaizen” system of pharmacotherapy. For example, we previously resolved drug–drug interaction between cefdinir prescribed after cataract surgery and magnesium oxide that a patient brought to the hospital by analyzing pharmaceutical interventions in the ophthalmology ward (postoperative antibiotics were changed from cefdinir to cefcapene pivoxil) [35]. Prevention of overdosage by linking a patient’s test results (e.g., eGFR and creatinine clearance) with prescriptions in an electronic health record system would also be beneficial. One limitation to our research was that pharmaceutical interventions were anonymously self-reported by pharmacists. For this reason, we could not explore the relationship between intervened DRPs and demographic information of facilities or pharmacists, or other factors. Nevertheless, our results provide useful information for understanding potential DRPs and actual pharmaceutical interventions in Japan, the most rapidly aging society in the world [36]. Our findings may also help to ensure safe and effective pharmacotherapy in Japan and other countries, in an era of aging populations.

## Conclusions

This study revealed potential DRPs by analyzing routine pharmaceutical interventions. Our results indicate that the analysis of routine pharmaceutical interventions may be useful for detecting potential DRPs. The contribution by pharmacists to providing safe medication and creating potential cost savings was also shown to be important in an aging society.

## Abbreviations

ADEs: Adverse drug events
ADRs: Adverse drug reactions
CKD: Chronic kidney disease
DRPs: Drug-related problems
eGFR: Estimated glomerular filtration rate
IQR: Interquartile range
MRSA: Methicillin-resistant *Staphylococcus aureus*
NSAIDs: Non-steroidal anti-inflammatory drugs
P-CAB: Potassium-competitive acid blocker
PIMs: Potentially inappropriate medications
PPI: Proton pump inhibitor
PT-INR: Prothrombin time-international normalized ratio
START: Screening Tool to Alert doctors to the Right Treatment
STOPP: Screening Tool of Older Person’s Prescriptions
TDM: Therapeutic drug monitoring
